# Regiochemical Control in a Thiol–Epoxy ‘Click’ Reaction: Synthesis of Cysteine and Glutathione Chain-End Functionalized Polyethylene Glycols

**DOI:** 10.3390/polym18141735

**Published:** 2026-07-15

**Authors:** Oana Grad, Crina Socaci, Mihaela Diana Lazar, Adrian Pîrnău, Anzar Khan

**Affiliations:** National Institute for Research and Development of Isotopic and Molecular Technologies—INCDTIM, 67-103 Donat Street, 400293 Cluj-Napoca, Romania; crina.socaci@itim-cj.ro (C.S.); diana.lazar@itim-cj.ro (M.D.L.); adrian.pirnau@itim-cj.ro (A.P.)

**Keywords:** ‘click’ reactions, polymer chain-end functionalization, amino-acid-containing polymers, cysteine-containing polymers, glutathione-containing polymers

## Abstract

The cysteine-based thiol–epoxy ‘click’ reaction is demonstrated as an efficient and practical approach for the synthesis of zwitterionic structures. The transformation employs unprotected cysteine, proceeds in aqueous media, and affords quantitative conversions. Notably, acid- and base-catalyzed conditions provide exclusive access to different cysteine-based thioether regioisomers in aqueous conditions. The pH-responsive behavior of the resulting zwitterions is further elucidated by NMR spectroscopy. Finally, the synthetic strategy is extended to the preparation of cysteine- and glutathione-functionalized polyethylene glycol polymers, showcasing its utility for the preparation of amino acid-/peptide-containing macromolecular materials.

## 1. Introduction

**Figure 1:**
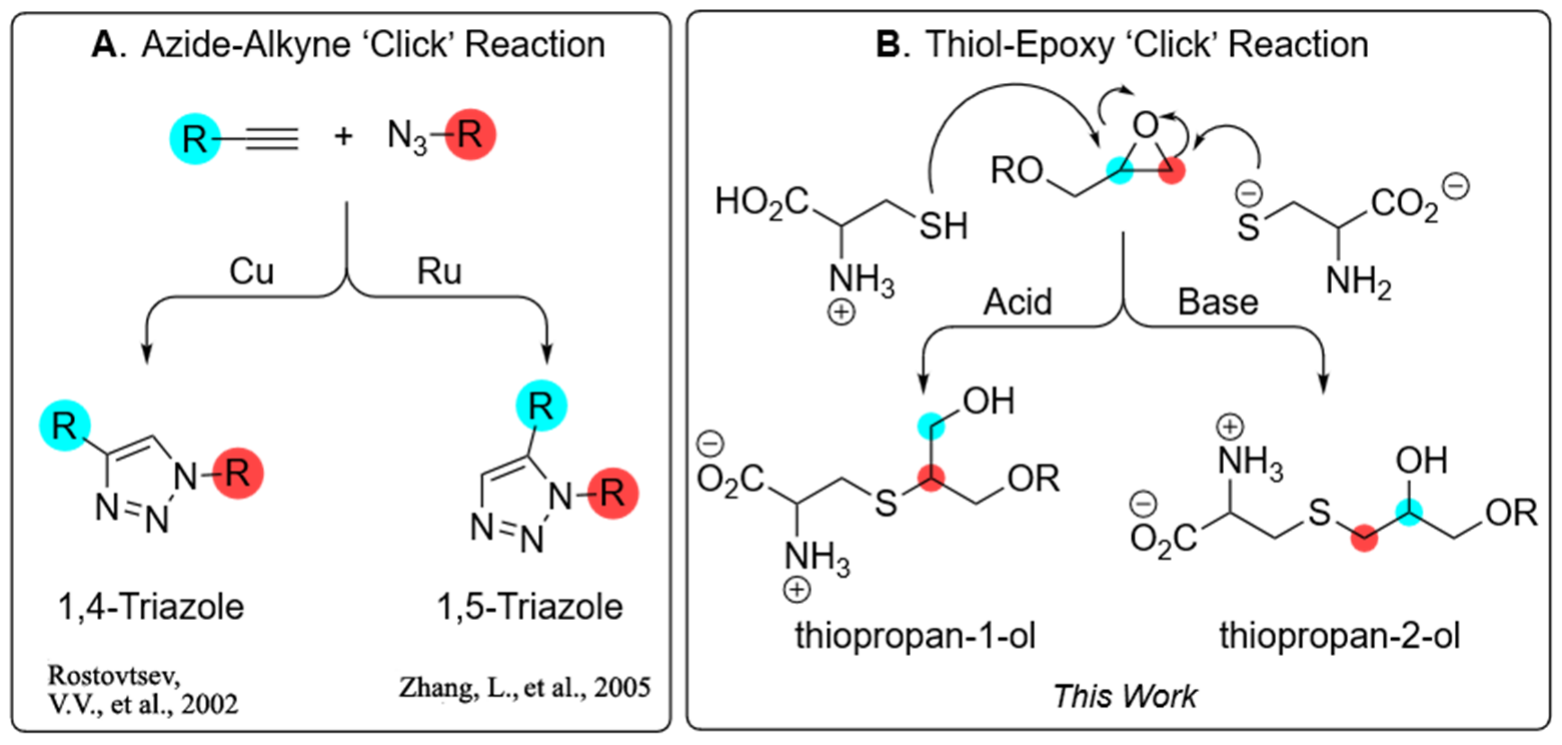
The copper and ruthenium-catalyzed ‘click’ reactions between azide and alkyne precursors to yield triazole regioisomers (**A**) [2,11]. The acid and base-catalyzed thiol–epoxy ‘click’ reaction leading to thioether regioisomers (**B**).

Regiochemical control is an important feature of many ‘click’ reactions and is central to their reliability in synthesis and material design [1,2,3,4,5,6,7,8,9,10]. The most significant example is the copper(I)-catalyzed azide–alkyne cycloaddition (CuAAC), which exclusively provides 1,4-disubstituted 1,2,3-triazole (Figure 1A) [1,2,3]. This contrasts with the uncatalyzed Huisgen cycloaddition that yields a mixture of 1,4- and 1,5-regioisomers [1]. This high level of regiochemical control arises from copper–acetylide formation and stepwise cycloaddition and underpins the widespread use of CuAAC in chemical biology, polymer chemistry, and surface functionalization [1,2,3]. To invert the regiochemical outcome, ruthenium-catalyzed azide–alkyne cycloaddition (RuAAC) can be applied, which selectively affords 1,5-disubstituted triazoles (Figure 1A) [11,12]. Regioisomerism in triazole-containing systems extends beyond a simple structural variation, as different triazole connectivities display distinct dipole moments, hydrogen-bonding characteristics, metal-coordination behavior, and electronic communication pathways. Consequently, triazole regiochemistry can significantly influence supramolecular organization, polymer thermal and electronic properties, and biological performance, including target binding and metabolic stability [12,13,14].

**Scheme 1:**
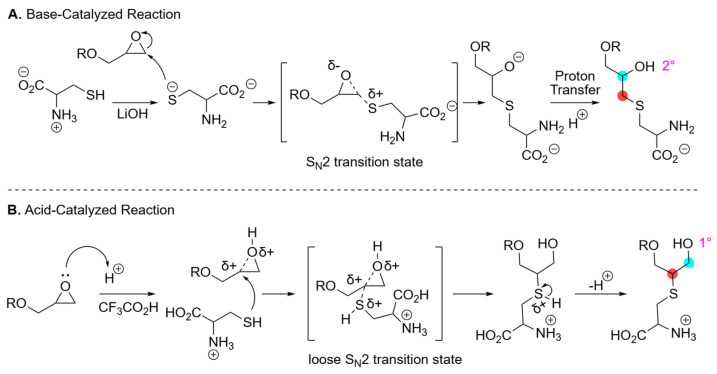
Plausible reaction mechanism for the base- (**A**) and acid- (**B**) catalyzed thiol–epoxy ‘click’ reactions.

Similar to the azide–alkyne reaction, thiol-based ‘click’ reactions such as thiol-ene and thiol-yne have been heavily influential in material synthesis [5,6,7,8,9]. In thiol-based chemistries, however, the preferred anti-Markovnikov addition cannot easily be switched to Markovnikov addition without losing the ‘click’ attributes of the reactions, such as product yields [5,6,7,8,9]. In considering other thiol-based ‘click’ reactions, thiol–epoxy reaction has found considerable applicability in the synthesis of functional soft materials [15]. The reaction employs strained three-membered epoxide rings as electrophiles and thiols as nucleophiles, resulting in the formation of thioether linkages through the epoxide ring-opening process. Due to its practicality, the reaction has found utility in the preparation of polymers and dendrimers [15,16,17,18], polyelectrolyte complexes [19], pressure-sensitive adhesives [20], functionalized polypeptides [21], self-assembling polymers [22], lectin-binding materials [23], drug-delivery materials [24,25], antibacterial materials [26], hydrogels [27,28,29], and fullerene-encapsulants [30]. Photocontrol over the reaction has also allowed for the development of photolithography applications [31,32]. A particularly interesting area is block copolymer lithography, in which the thiol–epoxy reaction plays a key role in attaching sidechains to copolymer precursors with a high propensity for phase-separation into nano-domains in thin films [33]. In all of these cases, only the base-catalyzed reaction is employed. In principle, the reaction can afford two regioisomers (Figure 1B and Scheme 1) [34]. However, thus far, a practical demonstration of this concept for quantitative, side-product-free ‘click’ transformations of unprotected amino acids in aqueous media remains elusive. Herein, we show that depending upon the nature of the reaction catalyst, regioselectivity can be governed in a thiol–epoxy ‘click’ reaction. The work is carried out using cysteine in its native form and the reactions are carried out in water. The pH-responsivity of the zwitterion is established with the help of NMR spectroscopy. The established concept can be applied to prepare cysteine and glutathione end-functional polyethylene glycols. In terms of cysteine, the prepared materials are zwitterionic in nature. The zwitterion-containing materials have found a very wide plethora of applications, ranging from antibiofouling surfaces to drug delivery, cell engineering, and bone remineralization [35]. However, to our knowledge, the examples of cysteine-based zwitterionic polymers remain limited [36,37]. Similarly, is the case of glutathione-containing polymers [37,38], despite their ability to cross the blood–brain barrier and the resulting potential for drug-delivery applications [38,39].

## 2. Materials and Methods

All the chemicals and solvents used in the synthetic protocols were purchased from commercial sources and used without further purification. The structures of the synthesized compounds were confirmed by ^1^H NMR and ^13^C NMR spectral analysis. The NMR spectra were recorded on an Advanced NMR spectrometer (Bruker, Bremen, Germany) at 500 MHz for ^1^H NMR and 125 MHz for ^13^C NMR, respectively. All chemical shifts (δ values) are reported in parts per million (ppm), and all coupling constants *J* are reported in Hz (using the D_2_O ^1^H peak as reference at 4.79 ppm). The designated multiplicities for the identified chemical shifts were abbreviated as follows: singlet (s), doublet of doublets (dd), triplet (t), multiplet (m). For ^1^H NMR, proton assignments were determined by COSY experiments, and, for ^13^C NMR, carbon assignments were determined by HMQC and HMBC experiments. The kinetics of the reaction were studied using a Thermo Fisher Scientific Vanquish HPLC (Germering, Germany) equipped with a Shim-pack GIST NH_2_ column (3 µm, 2.1 × 150 mm), maintained at 30 °C. The mobile phase consisted of isocratic elution with 0.1% formic acid in a water/acetonitrile mixture (70/30, *v*/*v*) at a flow rate of 0.20 mL/min. A DAD detector was set to 210 nm, allowing for the detection of ionized cysteine at 3.867 min. and the product at 3.096 min. during the analysis.

Reaction between cysteine and butyl glycidyl ether under basic conditions: L-cysteine (20 mg, 0.165 mmol, 1 eq) was placed in a screw-cap vial and dissolved in 2 mL of D_2_O. Butyl glycidyl ether (21.49 mg, 0.165 mmol, 1 eq) and LiOH (4 mg, 0.33 mmol, 2 eq) were then added while the mixture was maintained in an ice bath. The reaction mixture was left at room temperature, under a magnetic stirrer for 2 h. Subsequently, the reaction mixture was analyzed by NMR spectroscopy. ^1^H NMR (500 MHz, D_2_O) δ ppm: 3.87–3.92 (m, 1H, C*H*OH); 3.55–3.42 (m, 4H, C*H*_2_OC*H*_2_); 3.37–3.35 (m, 1H, C*H*(COO^−^)NH_2_); 2.85–2.68 (m, 2H, S-C*H*_2_-CH(COO^−^)NH_2_); 2.59–2.54 (m, 2H, C*H*_2_-CHOH); 1.53–1.47 (m, 2H, CH_2_); 1.32–1.25 (m, 2H, CH_2_), 0.84 (t, 3H, *J* = 7.4 Hz, CH_3_). ^13^C NMR (125 MHz, D_2_O) δ ppm: 180.98 (1C, COO^−^); 72.77 (1C, *C*H_2_-CHOH); 71.17 (1C, CH_2_); 68.95 (1C, CHOH); 55.16 (1C, *C*H(COO^−^)NH_2_); 37.55 (1C, S-*C*H_2_-CH(COO^−^)NH_2_); 35.19 (1C, *C*H_2_-CHOH); 30.62 (1C, CH_2_); 18.53 (1C, CH_2_);13.05 (1C, CH_3_).

Reaction between cysteine and butyl glycidyl ether under acidic conditions: L-cysteine (20 mg, 0.165 mmol, 1 eq) was placed in a screw-cap vial and dissolved in 2 mL of D_2_O. Butyl glycidyl ether (21.49 mg, 0.165 mmol, 1 eq) and TFA (75.25 mg, 0.66 mmol, 4 eq) were then added. The reaction mixture was left at room temperature, under a magnetic stirrer, overnight. Subsequently, the reaction mixture was analyzed by NMR spectroscopy. ^1^H NMR (500 MHz, D_2_O) δ ppm: 4.26–4.20 (m, 1H, -C*H*(COOH)NH_3_^+^); 3.71–3.75 (m, 1H, C*H-*CH_2_OH); 3.51–3.30 (m, 6H, CH*-*C*H*_2_OH, CHC*H*_2_OCH_2_, CHCH_2_OC*H*_2_); 3.02 (ddd, 2H, *J* = 19.4, 15.2, 4.9 Hz, S-C*H*_2_-CH(COOH)NH_3_^+^); 1.46–1.36 (m, 2H, CH_2_), 1.23–1.17 (m, 2H, CH_2_), 0.75 (t, *J* = 7.4 Hz, CH_3_). ^13^C NMR (125 MHz, D_2_O) δ ppm: 169.84 (1C, COOH); 72.77 (1C, *C*H_2_-CHOH); 71.10 (1C, CH*C*H_2_OCH_2_); 70.24 (1C, CHCH_2_O*C*H_2_); 62.60 (1C, CH*-C*H_2_OH); 54.16 (1C, *C*H(COOH)NH_3_^+^); 30.56 (1C, CH_2_); 23.66 (1C, *C*H_2_-CH(COOH) NH_3_^+^); 18.45 (1C, CH_2_); 12.98 (1C, CH_3_).

Chain-end functionalization of polyethylene glycol diglycidyl ether with cysteine: L-cysteine (20 mg, 0.165 mmol, 2 eq) was placed in a screw-cap vial and dissolved in 2 mL of D_2_O. PEG diglycidyl ether (41.25 mg, 0.085 mmol, 1 eq) and either LiOH (0.33 mmol, 4 eq) or TFA (0.66 mmol, 8 eq) were then added while maintaining the mixture in an ice bath. The reaction mixture was left at room temperature under a magnetic stirrer overnight. Subsequently, the reaction mixture was analyzed by NMR spectroscopy.

Cysteine-functionalized polyethylene glycol obtained under basic conditions: ^1^H NMR (500 MHz, D_2_O) δ ppm: 3.91 (bs, 2H, C*H*OH); 3.64 (s, 31H, CH_2_, PEG); 3.58–3.47 (m, 4H, C*H*_2_-CHOH); 3.36 (s, 2H, C*H*(COO^−^)NH_2_); 2.87–2.73 (m, 4H, S-C*H*_2_-CH(COO^−^)NH_2_); 2.63 (ddd, *J* = 21.2, 13.9, 6.3 Hz, 4H CHC*H*_2_O). ^13^C NMR (125 MHz, D_2_O) δ ppm: 180.91 (1C, COO^−^); 73.38 (1C, *C*H_2_-CHOH); 70.01 (1C, CH_2_–PEG); 69.53 (1C, CH_2_–PEG); 69.00 (1C, CHOH); 55.19 (1C, *C*H(COO^−^)NH_2_); 37.59 (1C, S-*C*H_2_-CH(COO^−^)NH_2_); 35.02 (1C, *C*H_2_-CHOH).

Cysteine-functionalized polyethylene glycol obtained under acidic conditions: ^1^H NMR (500 MHz, D_2_O) δ ppm: 3.88–3.9 (m, 1H, -C*H*(COOH)NH_3_^+^); 3.31 (bs, 2H, C*H-*CH_2_OH); 3.19 (s, 31H, CH_2_, PEG); 3.18–3.11 (m, 4H, CH*-*C*H*_2_OH); 3.10–3.00 (m, 4H, CHC*H*_2_O), 2.68 (qd, J = 15.2, 4.8 Hz, 4H, S-C*H*_2_-CH(COOH)NH_3_^+^). ^13^C NMR (125 MHz, D_2_O) δ ppm: 169.64 (1C, COOH); 71.41 (1C, *C*H_2_-CHOH); 70.15 (1C, CH*C*H_2_OCH_2_); 69.56 (1C, CH_2_–PEG); 69.38 (1C, CH_2_–PEG); 62.30 (1C, CH*-C*H_2_OH); 54.15 (1C, *C*H(COOH)NH_3_^+^); 23.55 (1C, *C*H_2_-CH(COOH)NH_3_^+^).

Chain-end functionalization of polyethylene glycol diglycidyl ether with glutathione: Glutathione (50.71 mg, 0.165 mmol, 2 equivalents) was placed in a screw-cap vial and dissolved in 2 mL of D_2_O. PEG diglycidyl ether (41.25 mg, 0.085 mmol, 1 equivalent) was then added, along with either LiOH (0.33 mmol, 4 equivalents) for basic conditions or TFA (0.66 mmol, 8 equivalents) for acidic conditions, while keeping the mixture in an ice bath. The reaction mixture was stirred overnight at room temperature using a magnetic stirrer. Afterward, the reaction mixture was analyzed by NMR spectroscopy.

Glutathione-functionalized polyethylene glycol obtained under basic conditions: ^1^H NMR (500 MHz, D_2_O) δ ppm: 3.9 (bs, 2H, C*H*OH); 3.60–3.7 (m, 2H, NHC*H*CO); 3.7 (s, 4H, NHC*H*_2_COOH); 3.64 (s, 31H, CH_2_, PEG); 3.56–3.45 (m, 4H, CHC*H*_2_O); 3.22–3.17 (m, 2H, C*H*(COO^−^)NH_2_); 3.09–2.80 (m, 4H, S-C*H*_2_-CHOH); 2.74–2.55 (m, 4H, NHCHC*H*_2_S); 2.34–2.27 (m, 4H, C*H*_2_CONH); 1.89–1.73 (m, 4H, C*H*_2_CH_2_CONH). ^13^C NMR (125 MHz, D_2_O) δ ppm: 182.47 (1C, COO^−^);176.36 (1C, COO^−^); 176.15 (1C, CO); 171.93 (1C, CO); 73.27 (1C, *C*H_2_-CHOH); 69.90 (1C, *C*H_2_OCH_2_); 71.50 (1C, CH_2_–PEG); 70.13 (1C, CH_2_–PEG); 69.29 (1C, CHOH); 68.88 (1C, *C*H(COO^−^)NH_2_); 43.27 (1C, *C*H_2_CH_2_CH(COO^−^)NH_2_); 34.86 (1C, CH_2_*C*H_2_CH(COO^−^)NH_2_); 33.49 (NHCH_2_COO^−^); 32.15 (1C, NHCH*C*H_2_S); 30.75 (1C, NH*C*HCH_2_S).

Glutathione-functionalized polyethylene glycol obtained under acidic conditions: ^1^H NMR (500 MHz, D_2_O) δ ppm: 4.41 (t, J = 6.1 Hz, 2H, NHC*H*CO); 3.97 (t, J = 6.6 Hz, 2H, C*H*(COOH)NH_3_^+^); 3.87 (s, 4H, NHC*H*_2_COOH); 3.72–3.64 (m, 2H, C*H-*CH_2_OH); 3.5 (s, 31H, CH_2_, PEG); 3.49–3.42 (m, 4H, CH*-*C*H*_2_OH); 3.41–3.55 (m, 4H, CHC*H*_2_O); 2.81–2.77 (m, 4H, NHCHC*H*_2_S); 2.51–2.42 (m. 4H, C*H*_2_CONH); 2.17–2.05 (m, 4H, C*H*_2_CH_2_CONH). ^13^C NMR (125 MHz, D_2_O) δ ppm: 174.21 (1C, COOH); 172.70 (1C, COOH); 171.35 (1C, CO); 171.23 (1C, CO); 71.60 (1C, CH*C*H_2_O); 70.21 (1C, *C*HCH_2_OH); 69.98 (1C, CH_2_–PEG); 69.44 (1C, CH_2_–PEG); 62.41 (1C, CH-*C*H_2_OH); 55.43 (1C, CH(COOH)NH_3_^+^); 51.94 (1C, NH*C*HCH_2_S); 40.99 (1C*C*H_2_CH_2_CH(COOH)NH_3_^+^); 30.65 (1C, CH_2_*C*H_2_CH(COOH)NH_3_^+^); 25.19 (1C, NHCH*C*H_2_S); 24.85 (NHCH_2_COOH).

## 3. Results and Discussion

The zwitterionic nature of cysteine suppresses nonspecific protein adsorption, and improves biocompatibility of polymer conjugates, thus making them useful at biointerfaces [40]. Covalent modification of cysteine in its unprotected (zwitterionic) form, however, is challenging due to the reactivity of the amine and acid groups. The only method to allow such orthogonal reactivity is based on the free radical thiol-ene chemistry which, as discussed above, does not allow control over regiochemistry. We envisaged that a higher reactivity of the thiolate anion (over amine and carboxylate nucleophiles) created under basic conditions could be employed to control chemoselectivity via a strict use of equimolar concentration of the reactants (Scheme 1A), while acidic conditions can be employed to quench the nucleophilic activity of the amine and the carboxylate groups via protonation (Scheme 1B). The reaction mechanism under basic and acidic conditions are anticipated to lead to two different regioisomers. If successful, such protocols can allow for cysteine conjugates to be prepared in a protective-group-free manner with regiochemical control.

**Figure 2:**
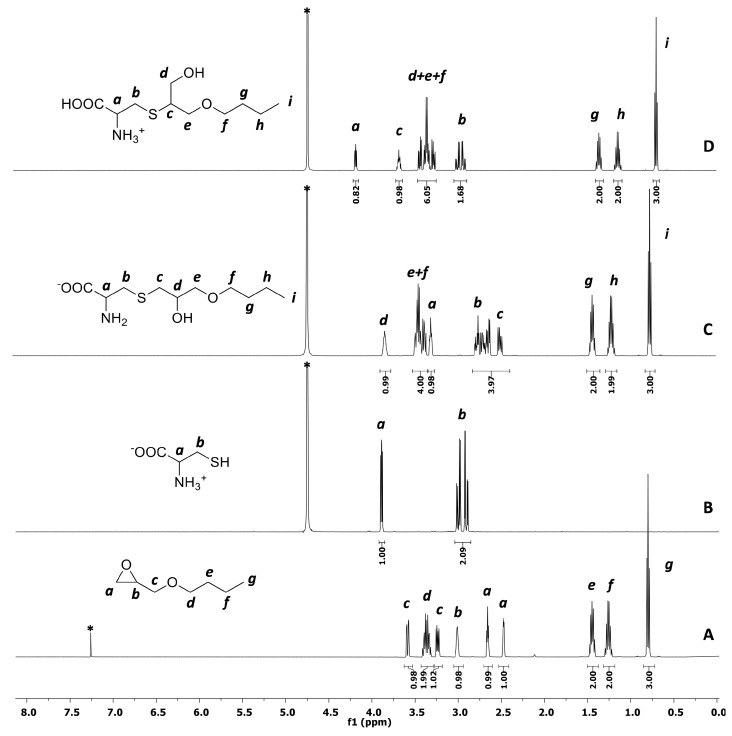
^1^H-NMR of glycidyl butyl ether (**A**), cysteine (under neutral conditions) (**B**), and the crude reaction mixtures in which the reactions are carried out under basic (**C**) and acidic (**D**) conditions. The residual solvent signals are shown with an asterisk.

To examine this hypothesis, initially, butyl glycidyl ether (Figure 2A) and cysteine (Figure 2B) were allowed to react in a 1:1 stoichiometry in the presence of various base catalysts in deuterated water, and the crude reaction mixtures were analyzed with the help of NMR spectroscopy (Figure 2C and Figures S1–S4). The oxirane ring-opening reaction was found to have conversions exceeding 90% while using one equivalent of triethylamine, 1,8-diazabicyclo[5.4.0]undec-7-ene, and lithium hydroxide as the base catalyst. The analysis of the crude reaction mixture was more practical with lithium hydroxide as it has no signal in the NMR spectrum. Thus, the later studies focused only on using lithium hydroxide. An increase in the lithium hydroxide content to two equivalents per thiol group led to a full conversion of the reactants into the anticipated thioether in two hours of reaction time.

**Figure 3:**
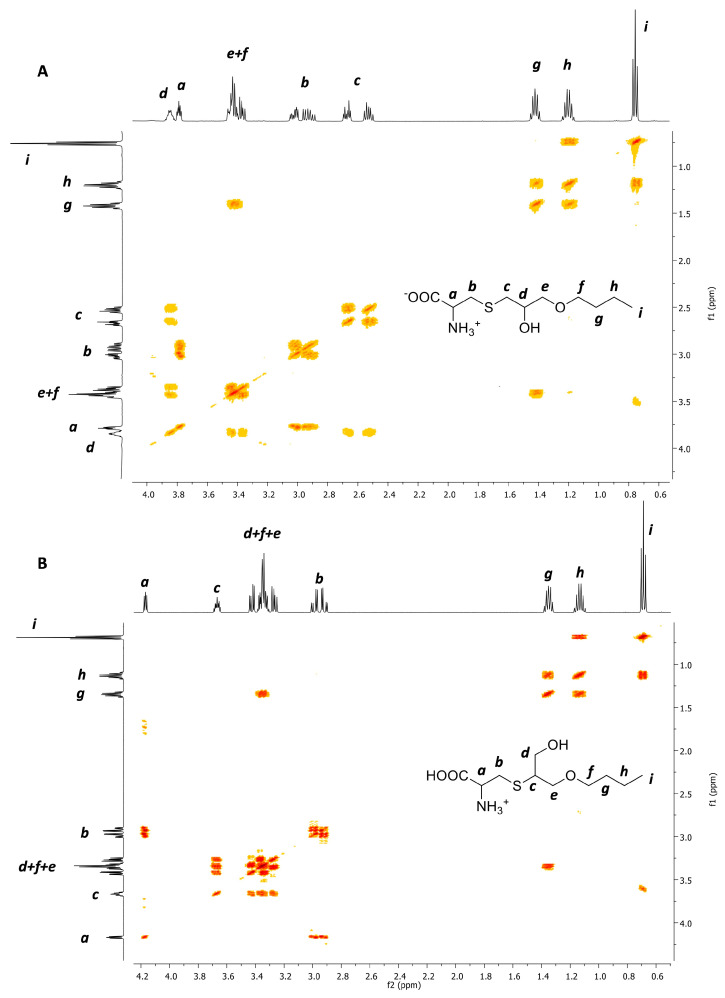
^1^H-^1^H COSY-NMR of the thioether regioisomers ((**A**) = obtained under basic conditions, (**B**) = obtained under acidic conditions) in deuterated water under neutral conditions at room temperature.

The molecular structure was determined with the help of 2D NMR spectroscopies (Figure 3A, Figures S5 and S6). The COSY spectrum exhibits a series of well-resolved cross-peaks that establish the expected interactions between protons located at the adjacent carbon atoms, as labeled in Figure 3. The observed correlations of the COSY spectrum, along with the HSQC and HMBC spectra, indicated that a secondary hydroxyl group had formed upon the ring-opening reaction, and that the new thioether bond was formed between the sulfur atom of cysteine and the least substituted carbon atom of the epoxide ring. This is in accordance with the known base-catalyzed thiol–epoxy reactions [34].

**Figure 4:**
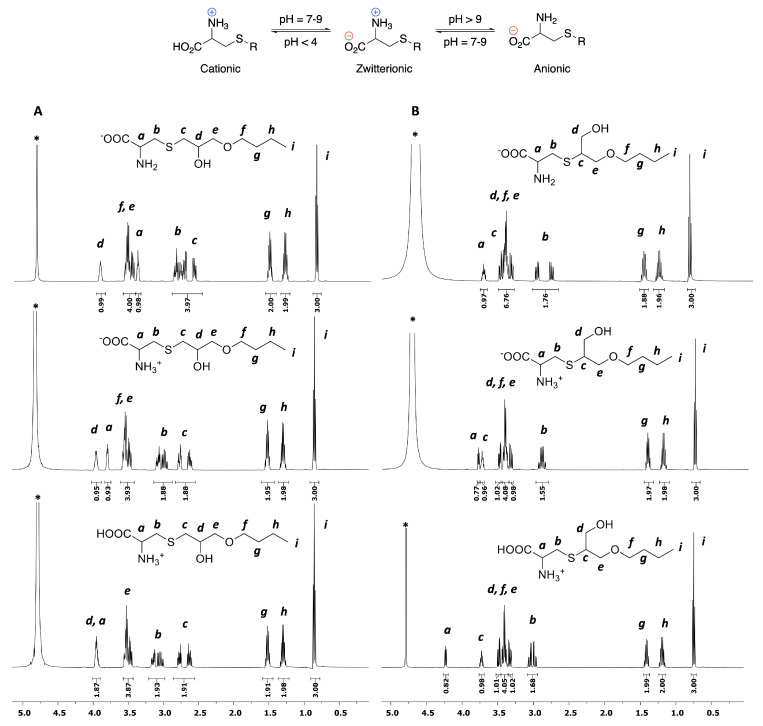
Sensitivity of cysteine-based thioethers towards changing pH conditions ((**A**) = isomer obtained under basic conditions, (**B**) = isomer obtained under acidic conditions). ^1^H-NMR of the thioether isomers under basic (**top**), neutral (**middle**), and acidic (**bottom**) conditions. The residual solvent signals are shown with an asterisk.

Further NMR experiments indicated that the zwitterion was intact and could be switched back and forth in a fully reversible manner into cationic, zwitterionic, and anionic forms by changing solution pH (Figure 4A, Figures S7 and S8). In all the cases, the signals corresponding to the protons in the alkyl chain appeared within the range of 0.8 to 1.6 ppm. Additionally, the proton resonances from the cysteine methine were detected at varying chemical shifts: 3.4 ppm for the anionic form, 3.89 ppm for the zwitterionic form, and 3.95 ppm for the cationic form (Figure 4A).

Next, a non-stoichiometric system was evaluated. For this, two equivalents of the epoxide were used with respect to cysteine. This reaction produced a complex product mixture, likely multi-substituted molecules through substitution at the amine site, indicating that the reaction is kinetically controlled, and other nucleophiles present in the system can also participate in the ring-opening reaction (Figure S9) [41]. Such non-stoichiometric systems can potentially be exploited in the synthesis of crosslinked materials (e.g., hydrogels) by using cysteine as an A_3_ monomer along with epoxide-based B_2_ monomers.

The attention was then shifted to the acidic conditions. For this, initially, acetic acid was used as the reaction medium. However, the reactants remained intact. Thus, TFA in dichloromethane was used, but led to homopolymerization of the butyl glycidyl ether through the ring-opening reaction. Finally, trifluoroacetic acid in water was used, which gave exclusively the second regioisomer. In this isomer, a primary hydroxyl group is formed, and the new thioether bond is established between the most substituted carbon atom of the epoxide ring and the sulfur atom of cysteine (Figure 2D, Figure 3B, Figures S10 and S11). The zwitterion remained intact and could be switched back and forth into the cationic and anionic structures, as also observed for the other regioisomer (Figure 4B). For the cationic structure (under acidic pH), the resonance from the cysteine methine appeared at 4.2 ppm. In the zwitterionic form (neutral pH), this resonance is observed at 3.9 ppm, about 0.3 ppm upfield, whereas in the anionic form (under basic pH), the resonance appeared at 3.8 ppm (Figure 4B and Figure S12). A similar trend is observed for the adjacent methylene group, which shifts from 3.1 ppm for the cationic form to 2.8 ppm for the anionic form. Interestingly, non-stoichiometric systems still led to a multi-substitution pattern, indicating that epoxide activation by trifluoroacetic acid played a key role in the reaction, as a weak nucleophile (amine under acidic conditions) could also open the ring successfully (Figure S13).

High pressure liquid chromatography (HPLC) analysis indicated that the reaction under basic conditions was fast, and the thiolate anion was consumed to a large extent within the first 22 min of the reaction time (Figure S14). Unfortunately, these aspects could not be examined under acidic conditions, due to the same retention time for cysteine and the corresponding thioether.

**Scheme 2:**
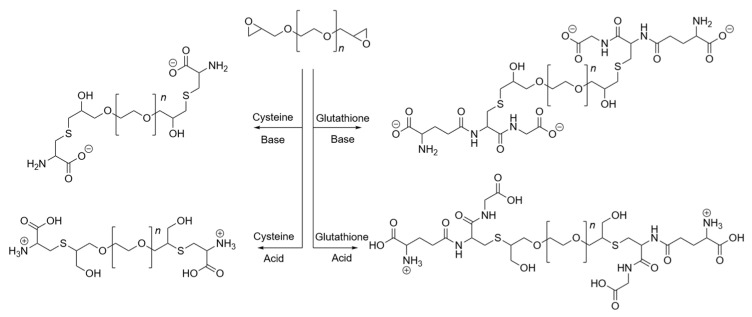
Post-synthesis modification of poly(ethylene glycol) diglycidyl ether with cysteine and glutathione.

**Figure 5:**
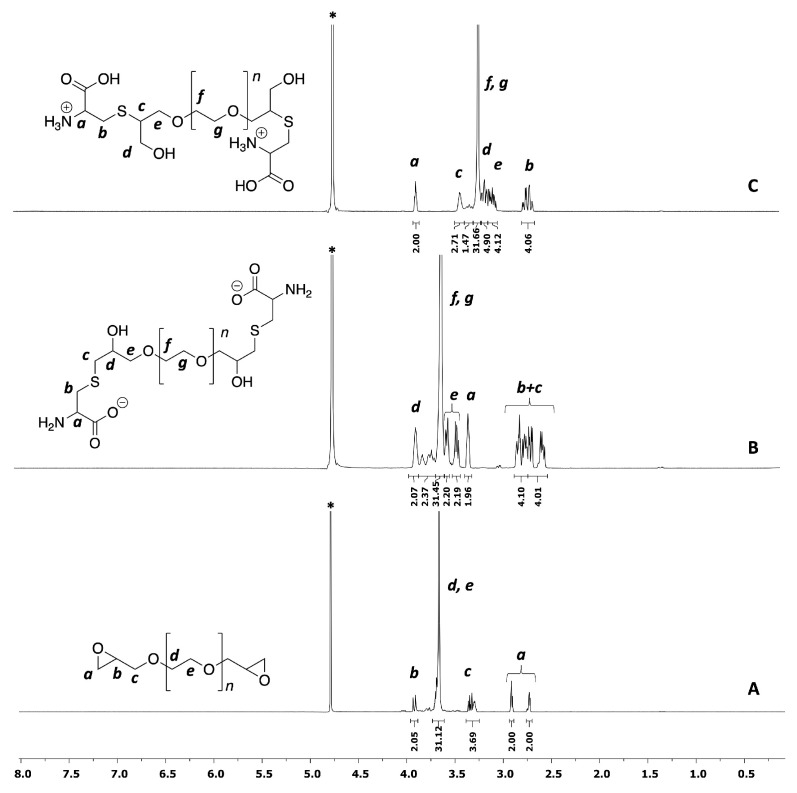
^1^H-NMR of the precursor polymer under neutral conditions (**A**) and crude reaction mixtures for reactions carried out under basic (**B**) and acidic (**C**) conditions. The residual solvent signals are shown with an asterisk.

The application aspects were then considered (Scheme 2). Amino-acid-containing synthetic polymers have emerged as a new materials platform in recent years [42]. Cysteine-containing polymers, however, remain rare [18,21]. Thus, commercial availability of polyethylene glycol with diglycidyl ether was exploited in the preparation of chain-end functional polymers (Figure 5, Figures S15 and S16). Chain-end functional polymers have also been recognized as important functional materials [43,44]. Poly(ethylene glycol) diglycidyl ether (*M*_n_ = 500) was subjected to the ring-opening reaction with cysteine under stoichiometric ratio. The NMR spectrum revealed proton resonances from the polymer backbone in the range of 3.1–3.3 ppm (Figure 5A). For the regioisomer obtained under basic conditions, the opening of the epoxide ring and the formation of the thioether bond are indicated by the appearance of a signal at 3.91 ppm, which corresponds to the methine proton (C*H*OH) of the alcohol group (Figure 5B). Additionally, a new resonance is observed around 3.59 ppm for the CH_2_ protons (C*H*_2_-CHOH), adjacent to the newly formed thioether linkage. For the regioisomer synthesized under acidic conditions, the methine proton (C*H*-S) appears upfield at 3.31 ppm (Figure 5C). This shift is attributed to its direct connection to the less electronegative sulfur atom. In comparison, the terminal methylene protons (*CH*_2_-OH) of the other regioisomer are shifted to a range of 3.18–3.11 ppm. The area integration analysis suggested a quantitative conversion of the glycidyl-functionalized polymer into a cysteine-functionalized macromolecular structure. The area integration analysis also indicated the polymer backbone remained intact, as is known for the thiol–epoxy reaction [15].

**Figure 6:**
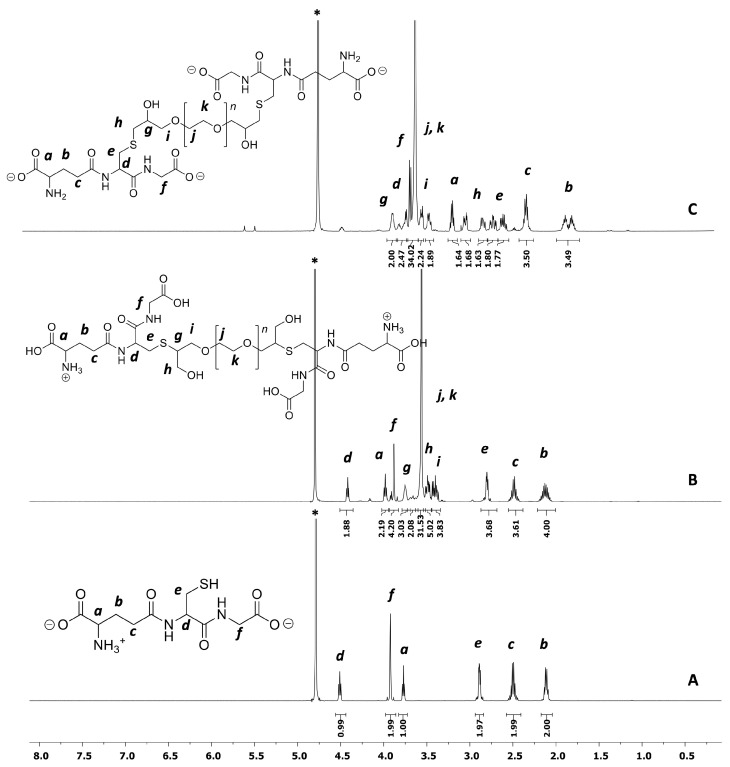
^1^H-NMR of glutathione under neutral conditions (**A**) and the crude reaction mixtures for reactions carried out under basic (**B**) and acidic (**C**) conditions. The residual solvent signals are shown with an asterisk.

Finally, a more challenging system, glutathione, was considered. Glutathione is a tripeptide, and its polymer conjugates are recognized to cross the blood–brain barrier (BBB) [38]. Thus, application of glutathione in the present design was tested and found to be successful, as new signals arising from the tripeptide could be located from 2.1 to 4.5 ppm (Figure 6A–C, Figures S17 and S18). The peptide conjugation reaction leads to the complete disappearance of the characteristic epoxide ring protons located in the region of 2.8–3.9 ppm. The successful formation of the thioether linkage is verified by a downfield shift in the methylene protons (C*H*_2_-S) of glutathione from 2.9 ppm to 3.1 ppm. Finally, area integration analysis in NMR spectroscopy suggests that the polymer backbone remains unperturbed.

In the present study, the synthesized polymers possessed relatively low molecular weights (*M*_n_ = 500 Da), which are below the molecular weight cut-off of conventional dialysis membranes (typically ≥1 kDa). As a result, dialysis was not a suitable method for polymer isolation. The use of higher-molecular-weight commercially available telechelic diglycidyl polymer precursors (*M*_n_ ≥ 1 kDa) would enable purification by dialysis in future studies. Nevertheless, to demonstrate the isolation of the functionalized polymers, the cysteine-functionalized polymer was selected as a model system, and a range of non-solvents was evaluated for precipitation. Several solvents produced only milky suspensions that could not be efficiently recovered by centrifugation. In contrast, precipitation in cold acetone (water:acetone = 1:20 (*v*/*v*)), followed by centrifugation and decantation of the supernatant, afforded the cysteine-functionalized polymer as a translucent material (Figure S19) in quantitative yield.

## 4. Conclusions

In summary, we have demonstrated that the cysteine-based thiol–epoxy click reaction provides a simple, efficient, and practical route to zwitterionic structures under protection-group-free conditions. The reaction proceeds in water, affords quantitative conversions, and, importantly, represents the first successful implementation of such pH-driven regiochemical control for the quantitative, side-product-free functionalization of unprotected cysteine in purely aqueous media. While base-catalyzed conditions selectively produce one thioether regioisomer via attack at the least-substituted carbon atom of the epoxide, acid-catalyzed conditions provide the complementary regioisomer resulting from attack at the more-substituted carbon atom. The resulting cysteine-derived zwitterions retain their pH-responsive behavior and can be reversibly interconverted between cationic, zwitterionic, and anionic forms. The synthetic utility of the present concept was further demonstrated through the preparation of cysteine- and glutathione-functionalized polyethylene glycol polymers. Given the growing interest in amino acid-containing polymers and the scarcity of glutathione-functionalized macromolecules, the developed synthetic strategy is expected to facilitate the design of new functional polymeric materials for applications at biointerfaces, in drug delivery, and in advanced biomaterials. Finally, establishing access to single regioisomers now provides a robust synthetic platform for future investigations into how regioisomerism influences the physical, chemical, and functional properties of the resulting polythioethers.

## Data Availability

The original contributions presented in this study are included in the article and Supplementary Material. Further inquiries can be directed to the corresponding authors.

## Supplementary Materials

The following supporting information can be downloaded at: https://www.mdpi.com/article/10.3390/polym18141735/s1, Figure S1: ^1^H NMR spectrum of cysteine under basic conditions in D_2_O at room temperature. Figure S2: ^1^H NMR spectrum of cysteine under acidic conditions in D_2_O at room temperature. Figure S3: ^1^H NMR of the two thioether isomers under neutral conditions in D_2_O at room temperature. The D_2_O solvent signal is shown with an asterisk. Figure S4: ^13^C NMR of cysteine-based thioether generated under basic and acidic conditions in D_2_O at room temperature. The residual solvent signals are shown with an asterisk. Figure S5: ^1^H-^13^C HSQC NMR of cysteine-based thioether generated under basic conditions in D_2_O at room temperature. Figure S6: ^1^H-^13^C HMBC NMR of cysteine-based thioether generated under basic conditions in D_2_O at room temperature. Figure S7: ^1^H NMR of the three forms of cysteine-based thioether generated under basic conditions in D_2_O at room temperature. The D_2_O solvent signal is shown with an asterisk. Figure S8: ^13^C NMR of the three forms of cysteine-based thioether generated under basic conditions in D_2_O at room temperature. Figure S9: ^1^H NMR of cysteine-based thioether with 1 eq. LiOH and 2 eq. of epoxide in D_2_O at room temperature. The D_2_O solvent signal is shown with an asterisk. Figure S10: ^1^H-^13^C HSQC NMR of cysteine-based thioether generated under acidic conditions in D_2_O at room temperature. Figure S11: ^1^H-^13^C HMBC NMR of cysteine-based thioether generated under acidic conditions in D_2_O at room temperature. Figure S12: ^1^H NMR of the three forms of cysteine-based thioether generated under acidic conditions in D_2_O at room temperature. The D_2_O solvent signal is shown with an asterisk. Figure S13: ^1^H NMR of cysteine-based thioether with 8 eq. of TFA and 2 eq. of epoxide in D_2_O at room temperature. The D_2_O solvent signal is shown with an asterisk. Figure S14: HPLC chromatograms showing consumption of cysteine and evolution of the reaction product as a function of reaction time. Figure S15: ^13^C NMR of cysteine-functionalized polyethylene glycol obtained under basic conditions in D_2_O at room temperature. Figure S16: ^13^C NMR of cysteine-functionalized polyethylene glycol obtained under acidic conditions in D_2_O at room temperature. The signals from trifluoroacetic acid are shown with an asterisk. Figure S17: ^13^C NMR of glutathione-functionalized polyethylene glycol obtained under basic conditions in D_2_O at room temperature. Figure S18: ^13^C NMR of glutathione-functionalized polyethylene glycol obtained under acidic conditions in D_2_O at room temperature. The signals from trifluoroacetic acid are shown with an asterisk. Figure S19: Digital picture of the isolated polymers in plastic centrifuge tubes.

## References

[1] Kolb H.C., Finn M.G., Sharpless K.B. (2001). Click Chemistry: Diverse Chemical Function from a Few Good Reactions. Angew. Chem. Int. Ed..

[2] Rostovtsev V.V., Green L.G., Fokin V.V., Sharpless K.B. (2002). A Stepwise Huisgen Cycloaddition Process: Copper(I)-Catalyzed Regioselective “Ligation” of Azides and Terminal Alkynes. Angew. Chem. Int. Ed..

[3] Hein J.E., Fokin V.V. (2010). Copper-Catalyzed Azide-Alkyne Cycloaddition (CuAAC) and beyond: New Reactivity of Copper(I) Acetylides. Chem. Soc. Rev..

[4] Tasdelen M.A., Yagci Y. (2013). Light-Induced Click Reactions. Angew. Chem. Int. Ed..

[5] Hoyle C.E., Bowman C.N. (2010). Thiol-Ene Click Chemistry. Angew. Chem. Int. Ed..

[6] Chan J.W., Hoyle C.E., Lowe A.B., Bowman M. (2010). Nucleophile-Initiated Thiol-Michael Reactions: Effect of Organocatalyst, Thiol, and Ene. Macromolecules.

[7] Fairbanks B.D., Scott T.F., Kloxin C.J., Anseth K.S., Bowman C.N. (2009). Thiol-Yne Photopolymerizations: Novel Mechanism, Kinetics, and Step-Growth Formation of Highly Cross-Linked Networks. Macromolecules.

[8] Kade M.J., Burke D.J., Hawker C.J. (2010). The Power of Thiol-Ene Chemistry. J. Polym. Sci. A Polym. Chem..

[9] Geng Z., Shin J.J., Xi Y., Hawker C.J. (2021). Click Chemistry Strategies for the Accelerated Synthesis of Functional Macromolecules. J. Polym. Sci..

[10] Lutz J.F., Zarafshani Z. (2008). Efficient Construction of Therapeutics, Bioconjugates, Biomaterials and Bioactive Surfaces Using Azide-Alkyne “Click” Chemistry. Adv. Drug Deliv. Rev..

[11] Zhang L., Chen X., Xue P., Sun H.H.Y., Williams I.D., Sharpless K.B., Fokin V.V., Jia G. (2005). Ruthenium-Catalyzed Cycloaddition of Alkynes and Organic Azides. J. Am. Chem. Soc..

[12] Johansson J.R., Beke-Somfai T., Said Stålsmeden A., Kann N. (2016). Ruthenium-Catalyzed Azide Alkyne Cycloaddition Reaction: Scope, Mechanism, and Applications. Chem. Rev..

[13] Agouram N., El Hadrami E.M., Bentama A. (2021). 1,2,3-Triazoles as Biomimetics in Peptide Science. Molecules.

[14] Ghorai A., Padmanaban E., Mukhopadhyay C., Achari B., Chattopadhyay P. (2012). Design and Synthesis of Regioisomeric Triazole Based Peptidomimetic Macrocycles and Their Dipole Moment Controlled Self-Assembly. Chem. Commun..

[15] Khan A. (2025). Thiol-Epoxy ‘Click’ Reaction in Polymer Synthesis. Prog. Polym. Sci..

[16] Stuparu M.C., Khan A. (2022). Poly (ß-hydroxy thioether)s: Synthesis through thiol-epoxy ‘click’reaction and post-polymerization modification to main-chain polysulfonium salts. J. Macromol. Sci. A.

[17] Stuparu M.C., Khan A. (2016). Thiol-epoxy “click” chemistry: Application in preparation and postpolymerization modification of polymers. J. Polym. Sci. Part A Polym. Chem..

[18] Gadwal I., Khan A. (2015). Multiply functionalized dendrimers: Protective-group-free synthesis through sequential thiol-epoxy ‘click’ chemistry and esterification reaction. RSC Adv..

[19] Stevens K.C., Tirrell M.V. (2024). Impact of a lightly branched star polyelectrolyte architecture on polyelectrolyte complexes. ACS Macro. Lett..

[20] Lee K., Tiu B.D., Martchenko V., Mai K., Lee G., Gerst M., Messersmith P.B. (2021). A modular strategy for functional pressure sensitive adhesives. ACS Appl. Mater. Interfaces.

[21] Perlin P., Scott W.A., Deming T.J. (2019). Modification of poly (5, 6-epoxy-l-norleucine) gives functional polypeptides with alternative side-chain linkages. Biomacromolecules.

[22] Howe D.H., Jenewein K.J., Hart J.L., Taheri M.L., Magenau A.J. (2020). Functionalization-induced self-assembly under ambient conditions via thiol-epoxide “click” chemistry. Polym. Chem..

[23] Oh T., Jono K., Kimoto Y., Hoshino Y., Miura Y. (2019). Preparation of multifunctional glycopolymers using double orthogonal reactions and the effect of electrostatic groups on the glycopolymer–lectin interaction. Polym. J..

[24] Porello I., Stucchi F., Sbaruffati G., Cellesi F. (2024). Tailoring copolymer architectures and macromolecular interactions for enhanced nanotherapeutic delivery: A design-by-architecture approach. Eur. Polym. J..

[25] Buerkli C., Lee S.H., Moroz E., Stuparu M.C., Leroux J.C., Khan A. (2014). Amphipathic homopolymers for siRNA delivery: Probing impact of bifunctional polymer composition on transfection. Biomacromolecules.

[26] Oh J., Khan A. (2021). Main-chain polysulfonium salts: Development of non-ammonium antibacterial polymers similar in their activity to antibiotic drugs vancomycin and kanamycin. Biomacromolecules.

[27] Cengiz N., Rao J., Sanyal A., Khan A. (2013). Designing functionalizable hydrogels through thiol–epoxy coupling chemistry. Chem. Commun..

[28] Gao L., Li X., Wang Y., Zhu W., Shen Z., Li X. (2016). Injectable thiol-epoxy “click” hydrogels. J. Polym. Sci. Part A Polym. Chem..

[29] Huynh C.T., Liu F., Cheng Y., Coughlin K.A., Alsberg E. (2018). Thiol-epoxy “click” chemistry to engineer cytocompatible PEG-based hydrogel for siRNA-mediated osteogenesis of hMSCs. ACS Appl. Mater. Interfaces.

[30] Eom T., Barát V., Khan A., Stuparu M.C. (2021). Aggregation-free and high stability core–shell polymer nanoparticles with high fullerene loading capacity, variable fullerene type, and compatibility towards biological conditions. Chem. Sci..

[31] Khan A. (2025). Photopolymerization Using Thiol–Epoxy ‘Click’ Reaction: Anionic Curing through Photolatent Superbases. ACS Polym. Au.

[32] Yeo H., Khan A. (2020). Photoinduced proton-transfer polymerization: A practical synthetic tool for soft lithography applications. J. Am. Chem. Soc..

[33] Feng H., Dolejsi M., Zhu N., Yim S., Loo W., Ma P., Zhou C., Craig G.S.W., Chen W., Wan L. (2022). Optimized design of block copolymers with covarying properties for nanolithography. Nat. Mater..

[34] Khan A. (2023). Thiol-Epoxy ‘Click’ Chemistry: A Focus on Molecular Attributes in the Context of Polymer Chemistry. Chem. Commun..

[35] Wang H., Cheng K., Zheng H., Zhao X., Chen D., Tian X., Sun J., Cheng Y., Lang L., Bao S. (2026). Zwitterionic Polymers: Synthesis, Architectures, Properties and Biomedical Applications. Adv. Mater..

[36] Ladmiral V., Charlot A., Semsarilar M., Armes S.P. (2015). Synthesis and Characterization of Poly(Amino Acid Methacrylate)-Stabilized Diblock Copolymer Nano-Objects. Polym. Chem..

[37] Alswieleh A.M., Cheng N., Canton I., Ustbas B., Xue X., Ladmiral V., Xia S., Ducker R.E., El Zubir O., Cartron M.L. (2014). Zwitterionic Poly(amino acid methacrylate) Brushes. J. Am. Chem. Soc..

[38] Englert C., Trützschler A.K., Raasch M., Bus T., Borchers P., Mosig A.S., Traeger A., Schubert U.S. (2016). Crossing the Blood-Brain Barrier: Glutathione-Conjugated Poly(Ethylene Imine) for Gene Delivery. J. Control. Release.

[39] Guan X. (2024). Glutathione Transporter as a Target for Brain Drug Delivery. Med. Chem. Res..

[40] Ahangarpour M., Kavianinia I., Brimble M.A. (2023). Thia-Michael Addition: The Route to Promising Opportunities for Fast and Cysteine-Specific Modification. Org. Biomol. Chem..

[41] Saha A., De S., Stuparu M.C., Khan A. (2012). Facile and General Preparation of Multifunctional Main-Chain Cationic Polymers through Application of Robust, Efficient, and Orthogonal Click Chemistries. J. Am. Chem. Soc..

[42] Nayak K., Ghosh P., Khan M.E.H., De P. (2022). Side-Chain Amino-Acid-Based Polymers: Self-Assembly and Bioapplications. Polym. Int..

[43] Lunn D.J., Discekici E.H., Read de Alaniz J., Gutekunst W.R., Hawker C.J. (2017). Established and Emerging Strategies for Polymer Chain-End Modification. J. Polym. Sci. A Polym. Chem..

[44] Lee H., Lee Y., Kim N., Park M.J. (2025). Polymer Chain-End Chemistry: Unlocking next-Generation Functional Materials. Prog. Polym. Sci..

