# Supplementary material for: Regiochemical Control in a Thiol–Epoxy ‘Click’ Reaction: Synthesis of Cysteine and Glutathione Chain-End Functionalized Polyethylene Glycols
Source: Polymers (Basel). 2026 Jul 15;18(14):1735. doi: 10.3390/polym18141735 (PMC13417147; doi:10.3390/polym18141735)
Supplement: Supplementary file 1 [file polymers-18-01735-s001.zip › polymers-4394876-supplementary.pdf]

Supporting Information

Regiochemical Control in a Thiol-Epoxy 'Click' Reaction: Synthesis of Cysteine and Glutathione Chain-End Functionalized Polyethylene Glycols

Oana Grad,* Crina Socaci, Mihaela Diana Lazar, Adrian Pîrnău, Anzar Khan*

National Institute for Research and Development of Isotopic and Molecular Technologies - INCDTIM, 67-103 Donat Street, 400293 Cluj-Napoca, Romania

*Correspondence: oana.grad@itim-cj.ro; akhan@itim-cj.ro

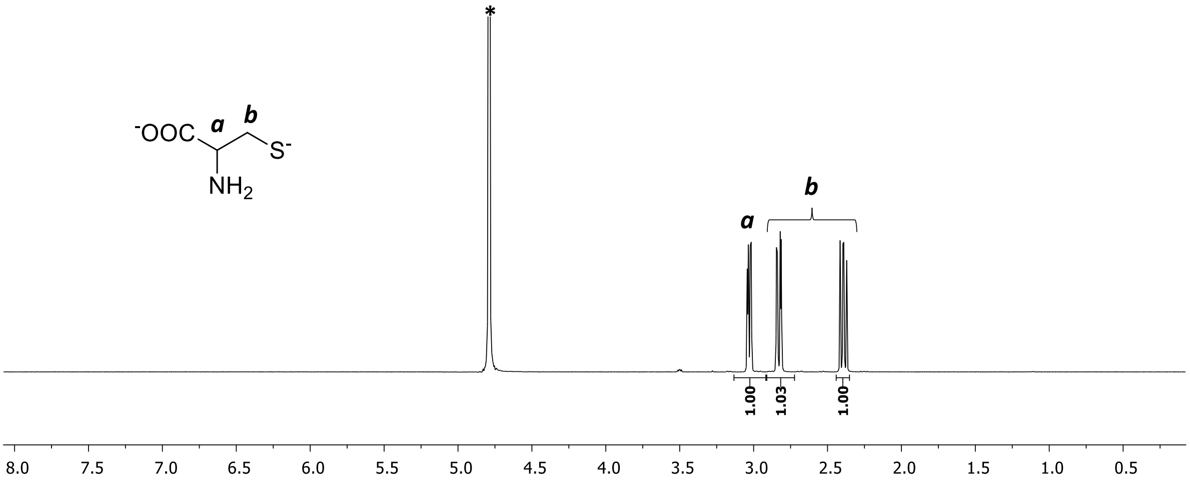

Figure S1. ^{1}H NMR spectrum of cysteine in its thiolate/carboxylate form under basic conditions in D_2O at room temperature.

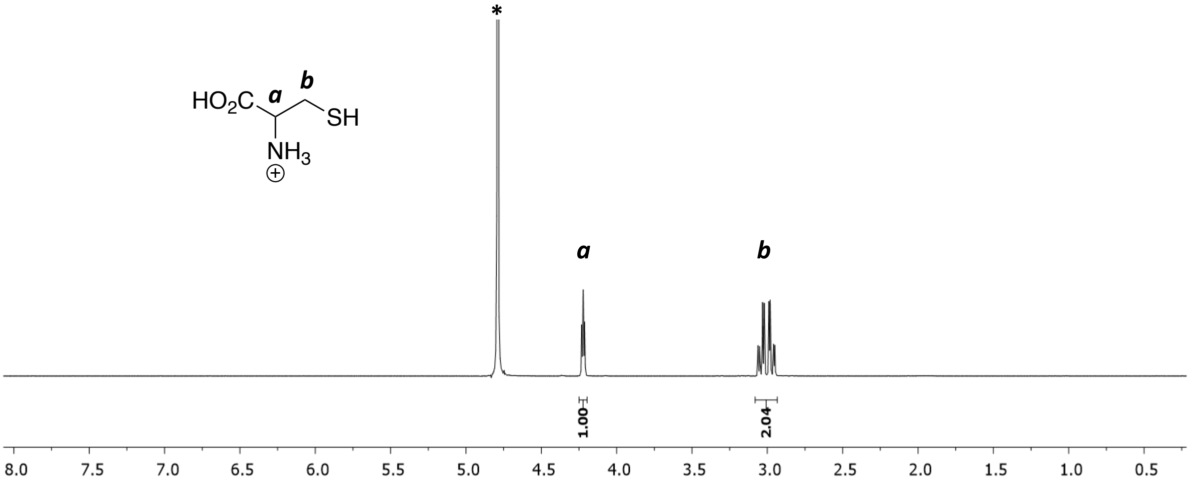

Figure S2. ^{1}H NMR spectrum of fully protonated cysteine under acidic conditions in D_2O at room temperature.

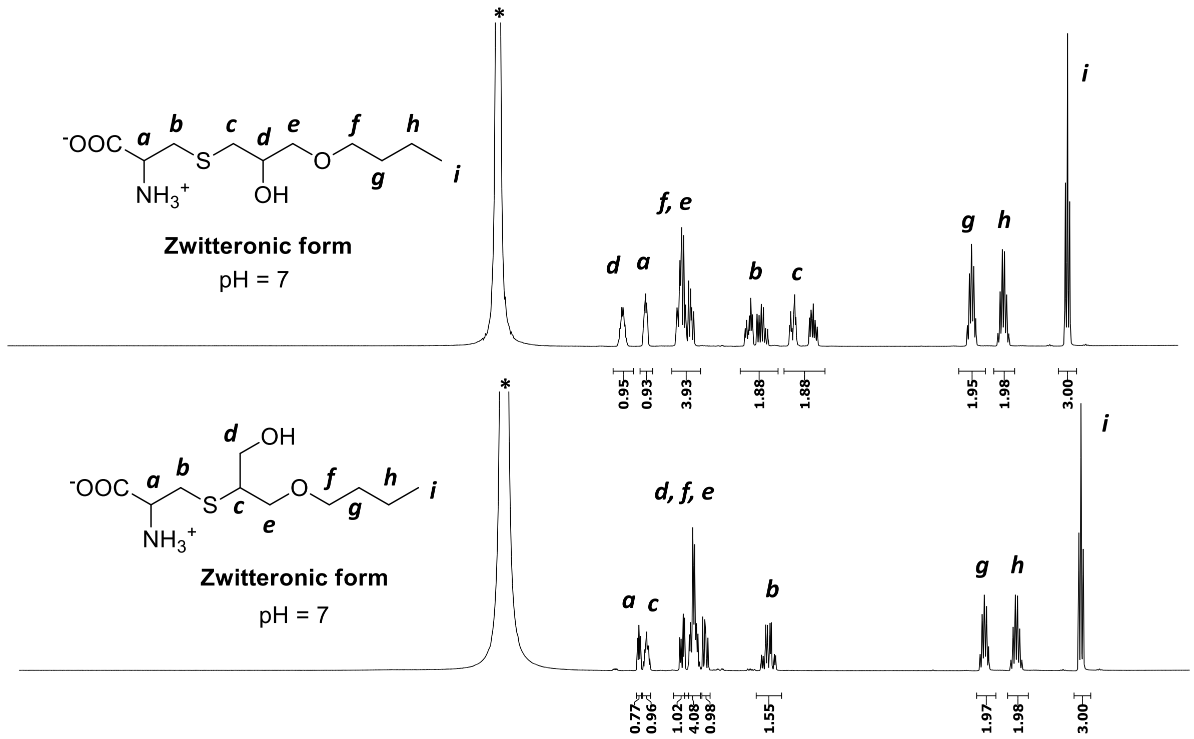

Figure S3. ^{1}H NMR of the two thioether isomers under neutral conditions in D_2O at room temperature. The D_2O solvent signal is shown with an asterisk.

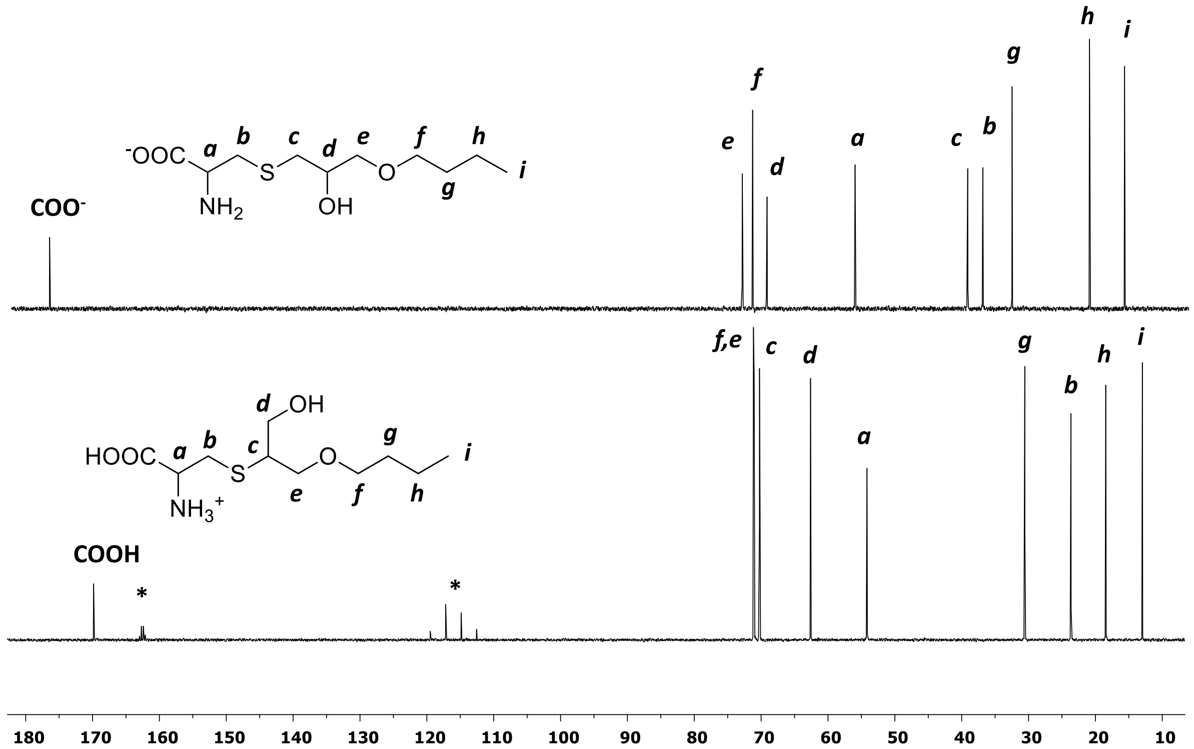

Figure S4. ^{13}C NMR of cysteine-based thioether generated under basic and acidic conditions in D_2O at room temperature. The residual solvent signals are shown with an asterisk.

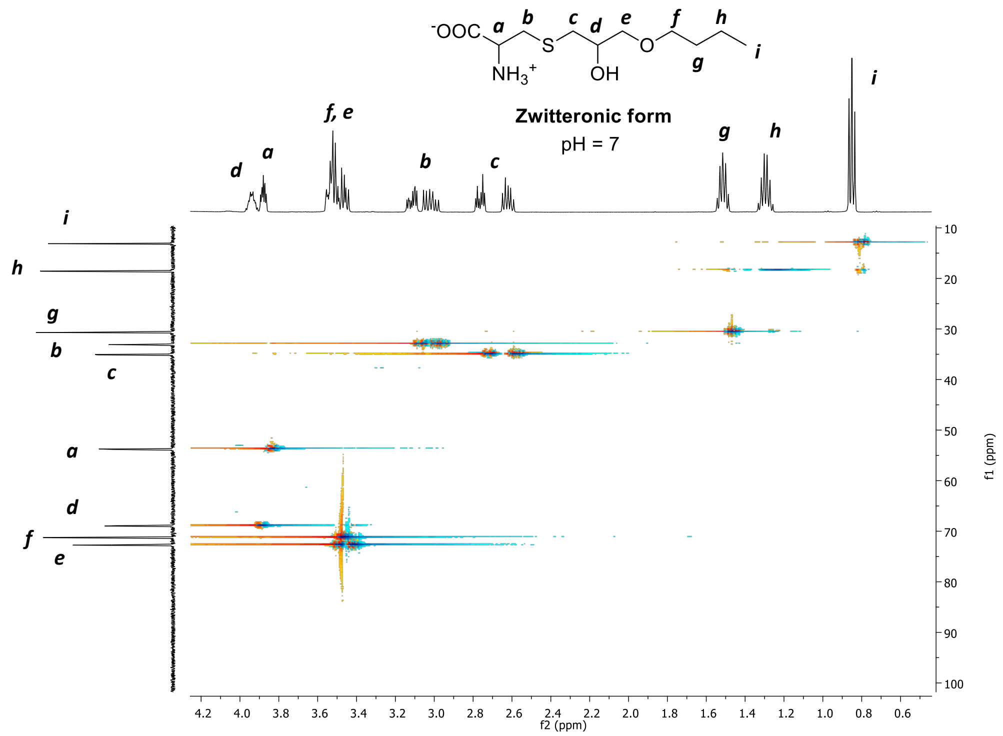

Figure S5. ^{1}H-^{13}C HSQC NMR of cysteine-based thioether generated under basic conditions in D_2O at room temperature.

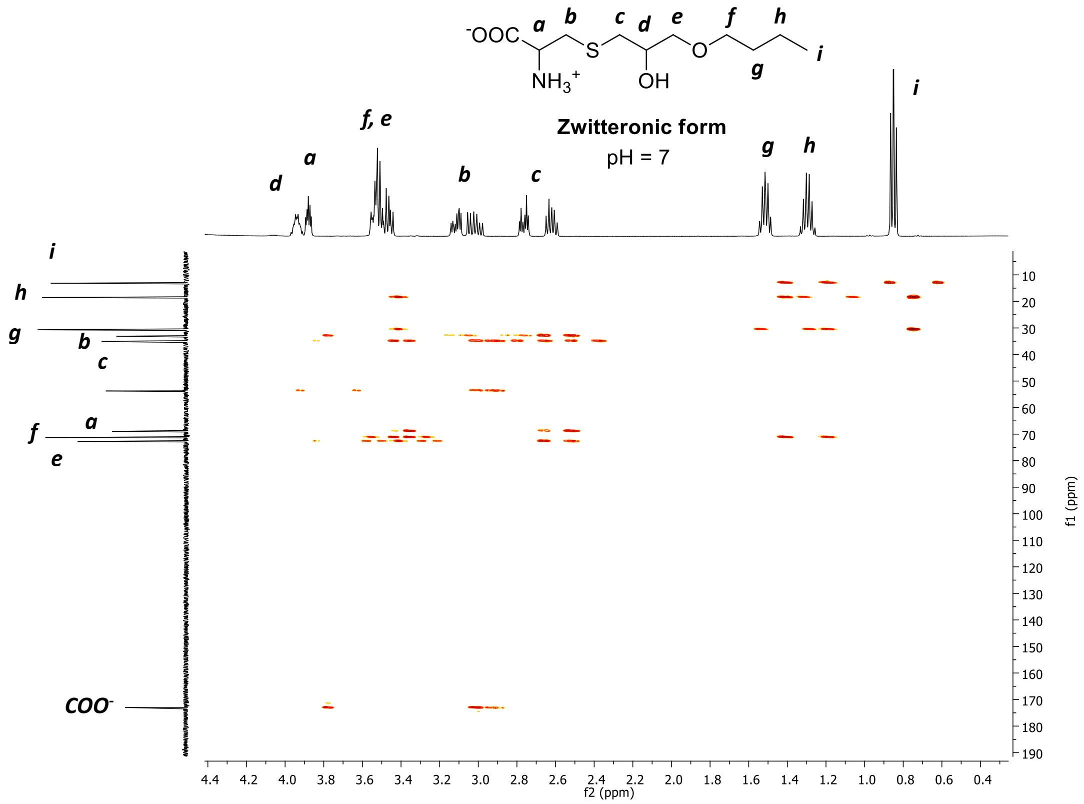

Figure S6. ^{1}H-^{13}C HMBC NMR of cysteine-based thioether generated under basic conditions in D_2O at room temperature.

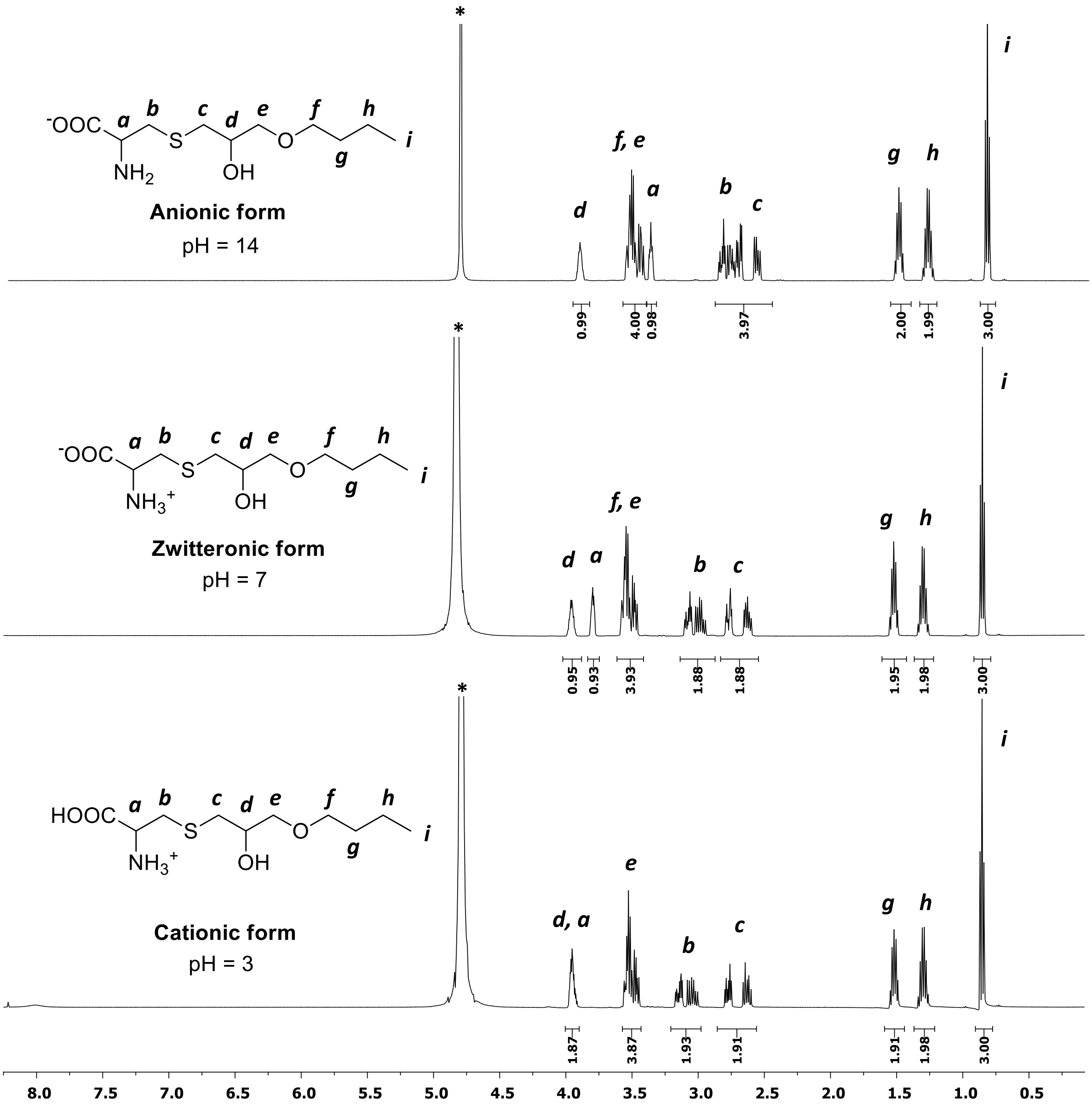

Figure S7. ^{1}H NMR of the three forms of cysteine-based thioether generated under basic conditions in D_2O at room temperature. The D_2O solvent signal is shown with an asterisk.

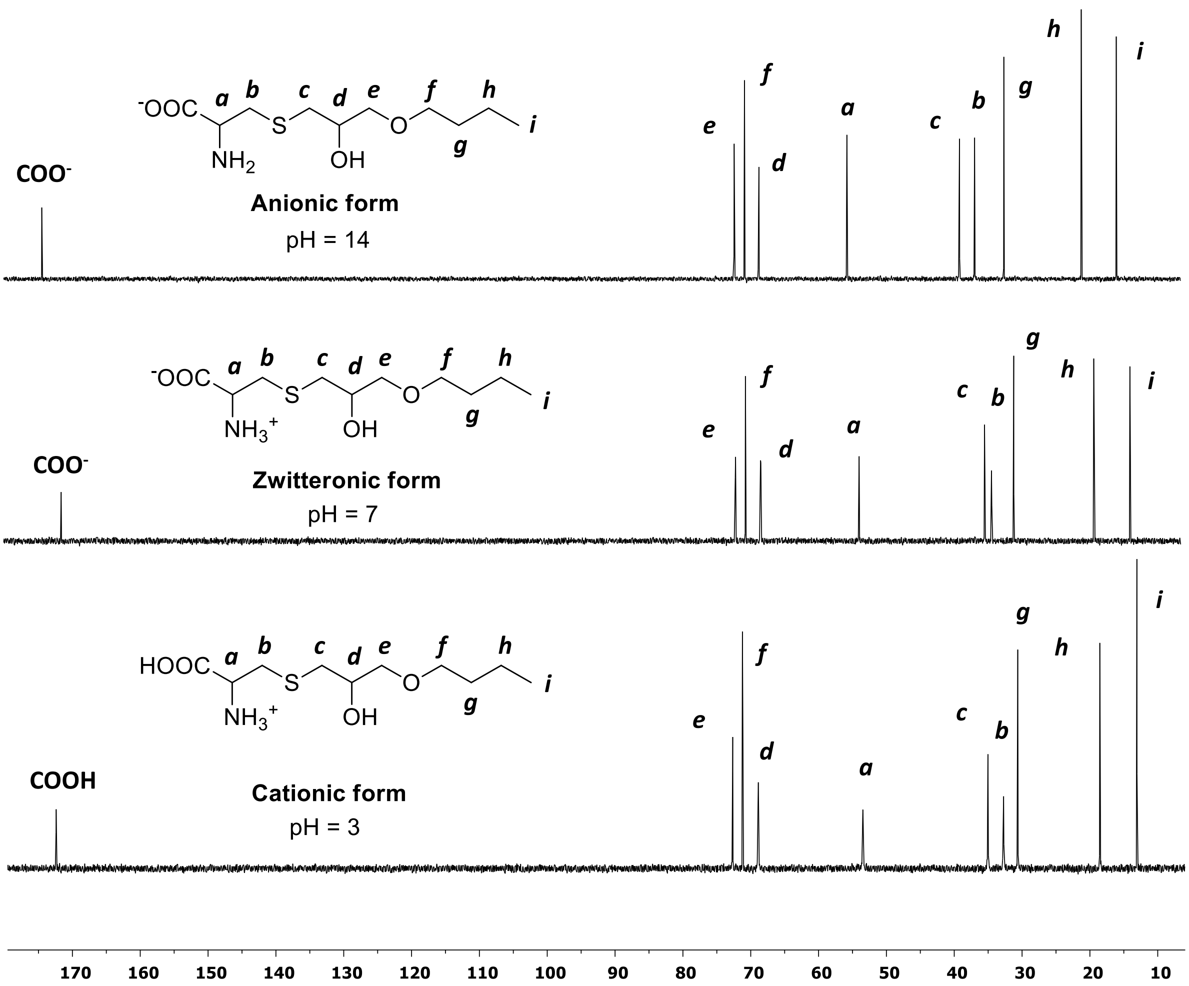

Figure S8. ^{13}C NMR of the three forms of cysteine-based thioether generated under basic conditions in D_2O at room temperature.

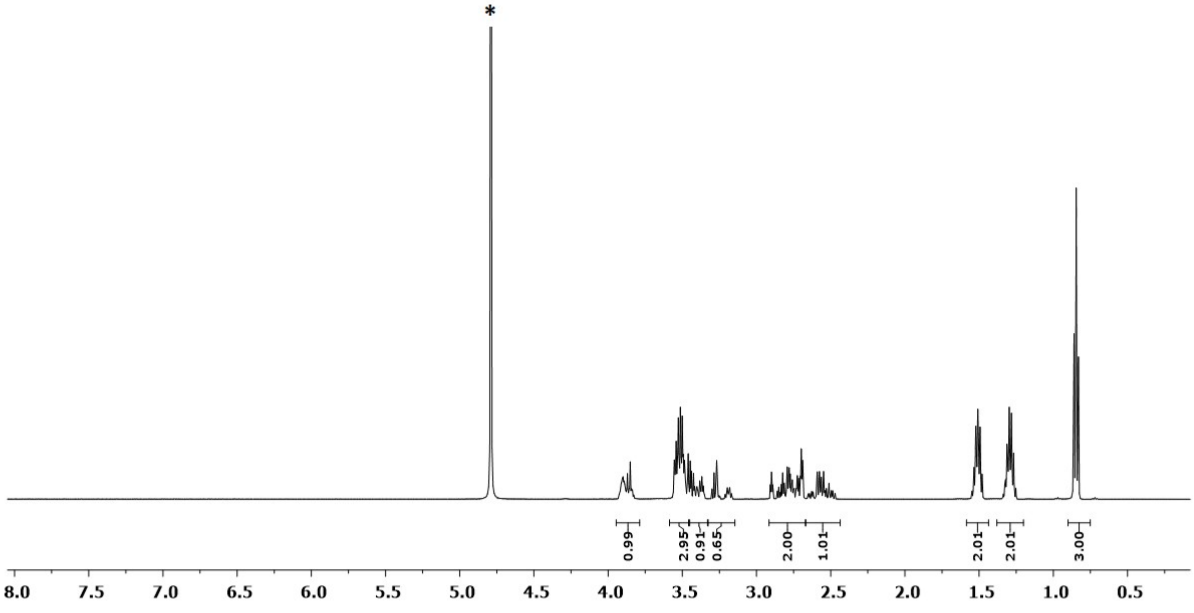

Figure S9. ^{1}H NMR of cysteine-based thioether with 1 eq. LiOH and 2 eq. of epoxide in D_2O at room temperature. The D_2O solvent signal is shown with an asterisk.

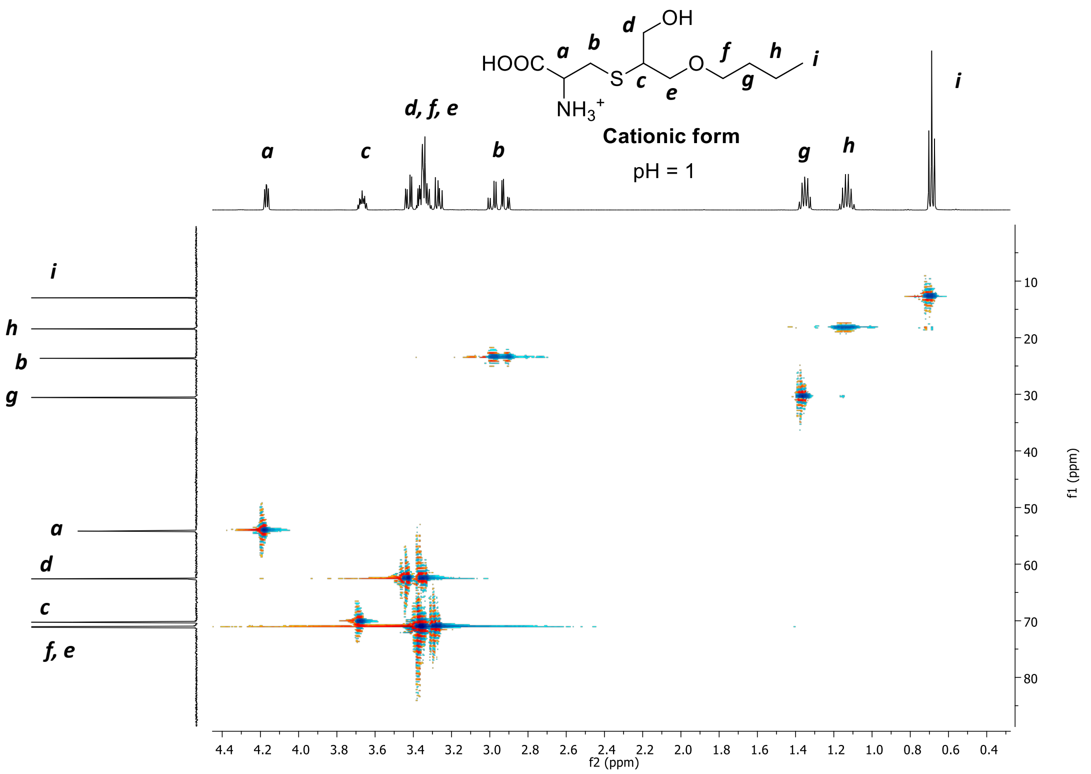

Figure S10. ^{1}H-^{13}C HSQC NMR of cysteine-based thioether generated under acidic conditions in D_2O at room temperature.

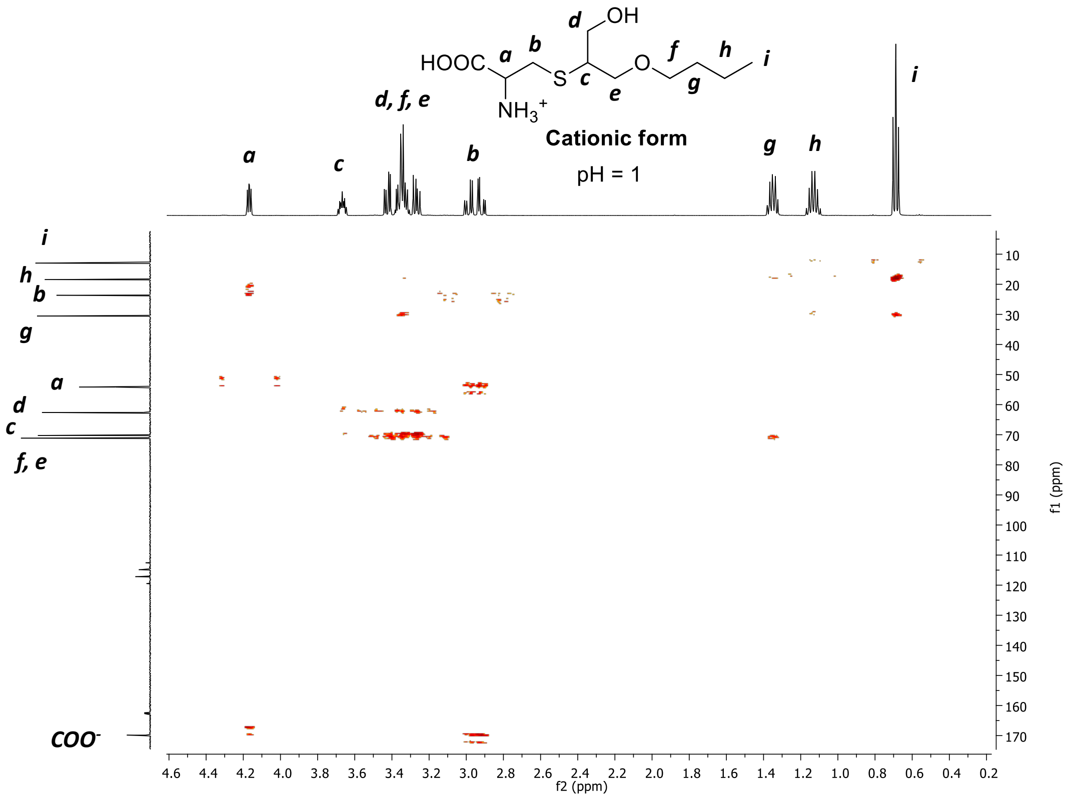

Figure S11. ^{1}H-^{13}C HMBC NMR of cysteine-based thioether generated under acidic conditions in D_2O at room temperature.

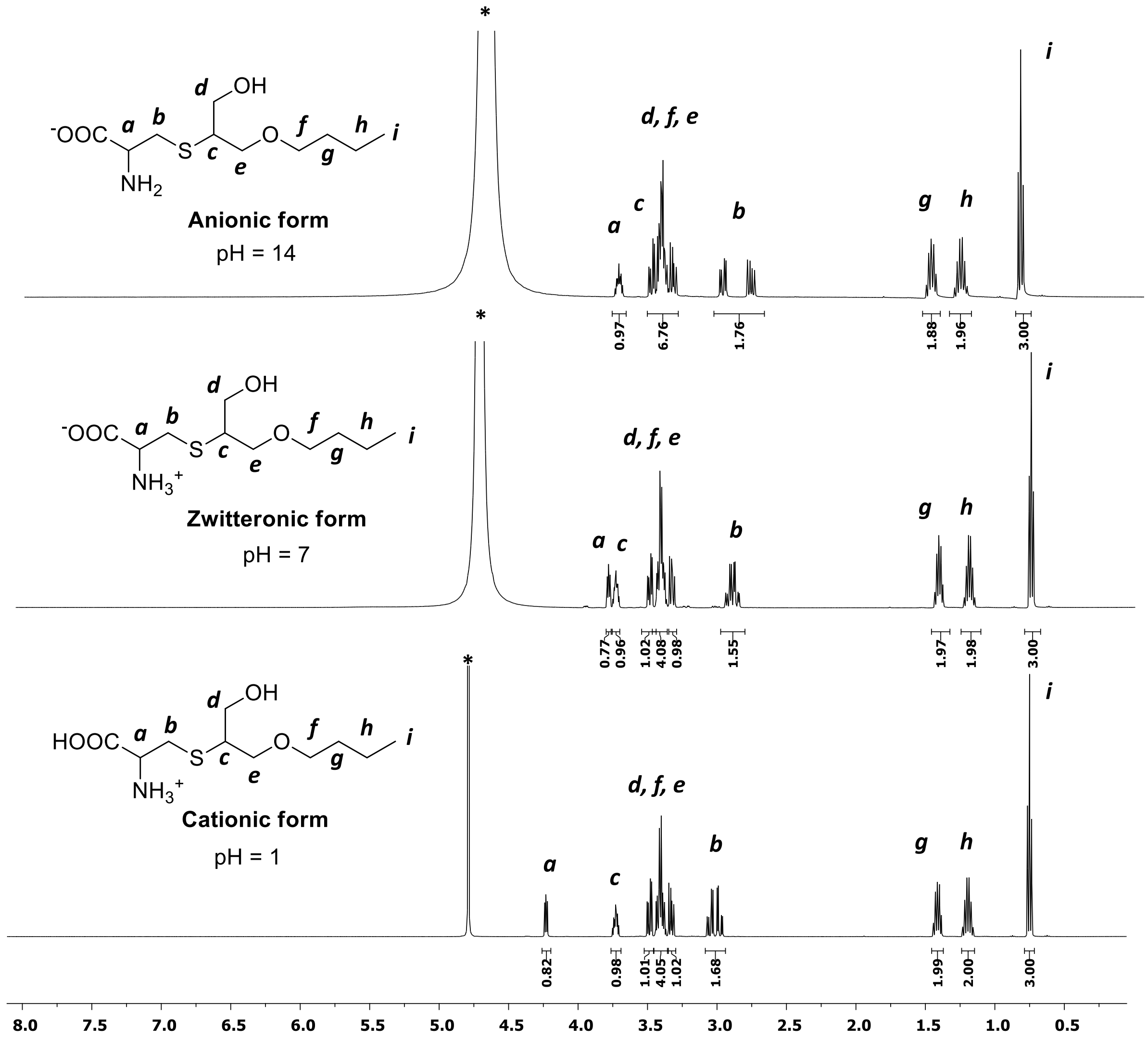

Figure S12. ^{1}H NMR of the three forms of cysteine-based thioether generated under acidic conditions in D_2O at room temperature. The D_2O solvent signal is shown with an asterisk.

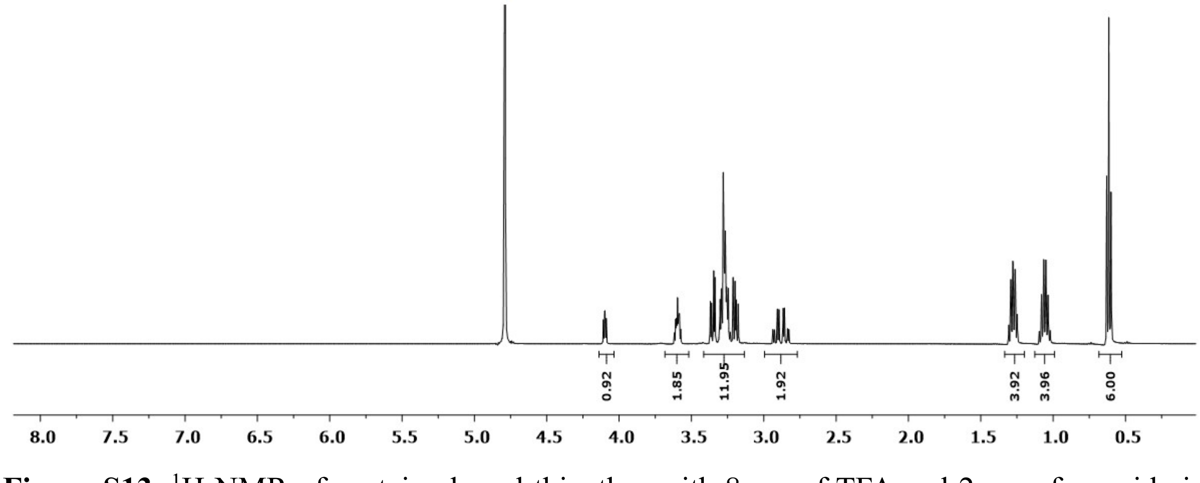

Figure S13. ^{1}H NMR of cysteine-based thioether with 8 eq. of TFA and 2 eq. of epoxide in D_2O at room temperature. The D_2O solvent signal is shown with an asterisk.

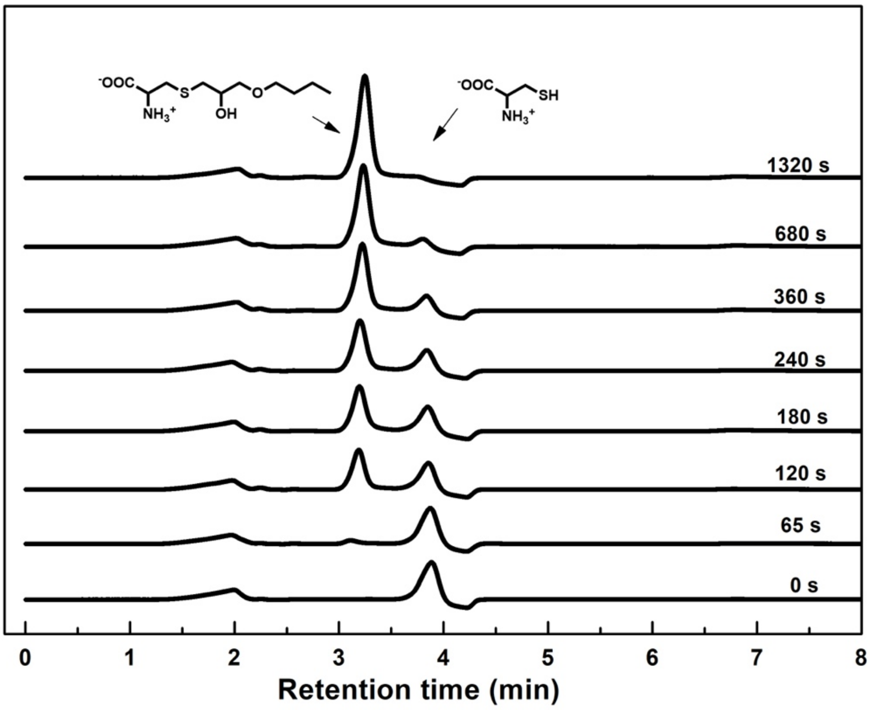

Figure S14. HPLC chromatograms showing consumption of cysteine and evolution of the reaction product as a function of reaction time.

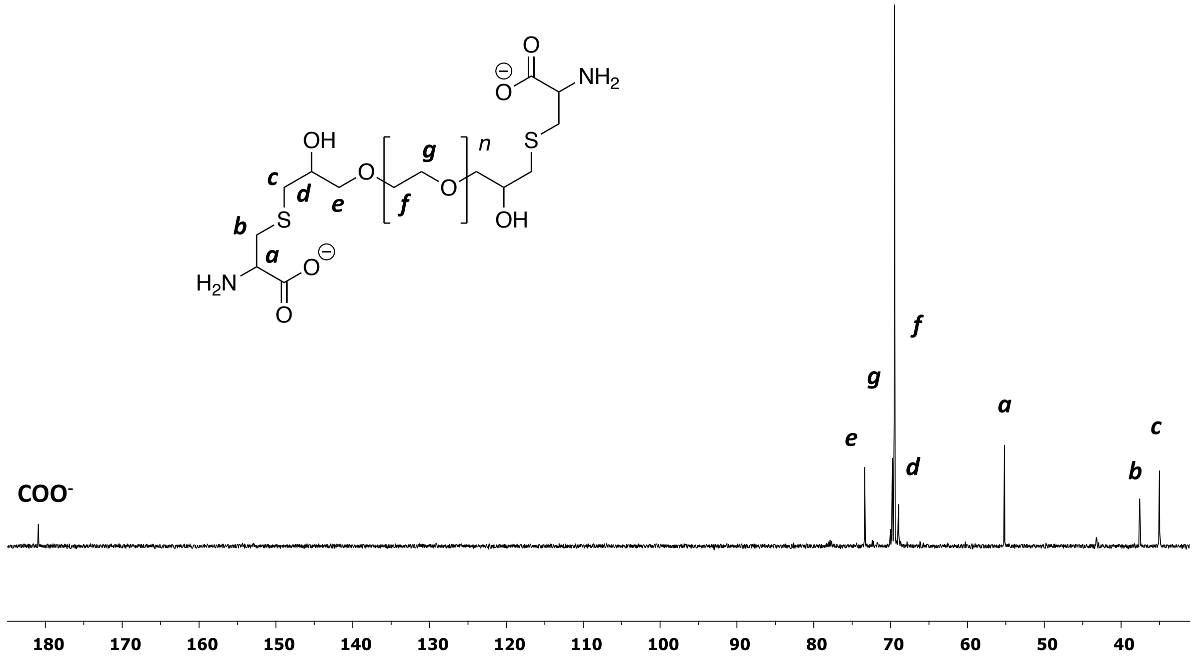

Figure S15. ^{13}C NMR of cysteine-functionalized polyethylene glycol obtained under basic conditions in D_2O at room temperature.

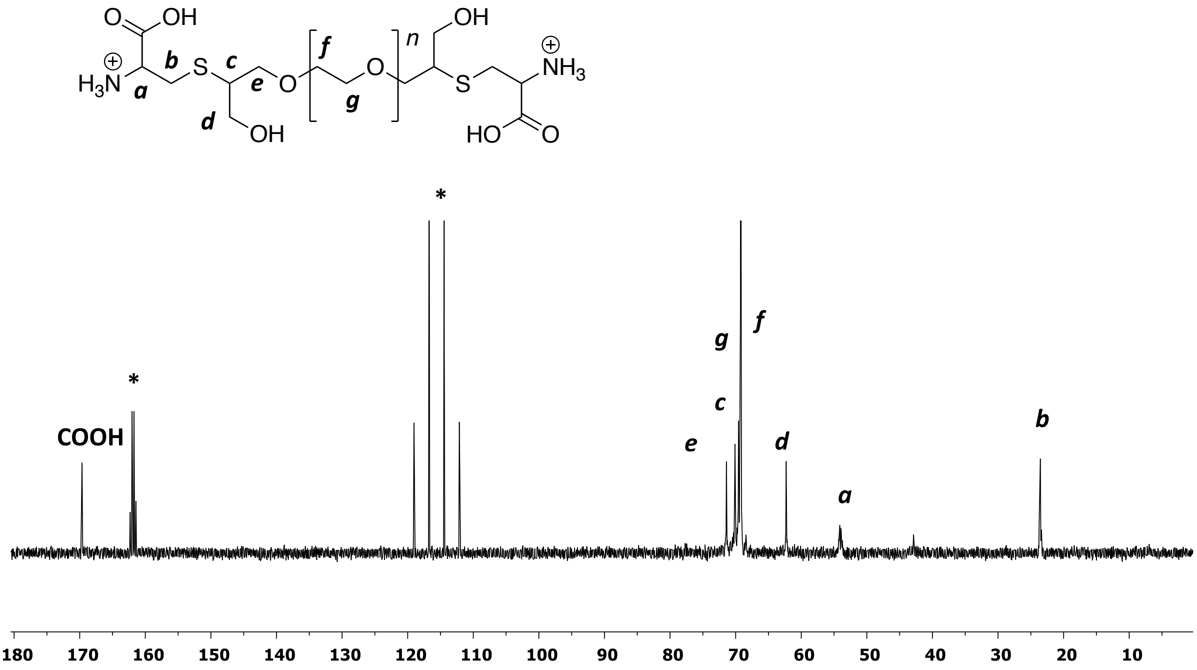

Figure S16. ^{13}C NMR of cysteine-functionalized polyethylene glycol obtained under acidic conditions in D_2O at room temperature. The signals from trifluoroacetic acid are shown with an asterisk.

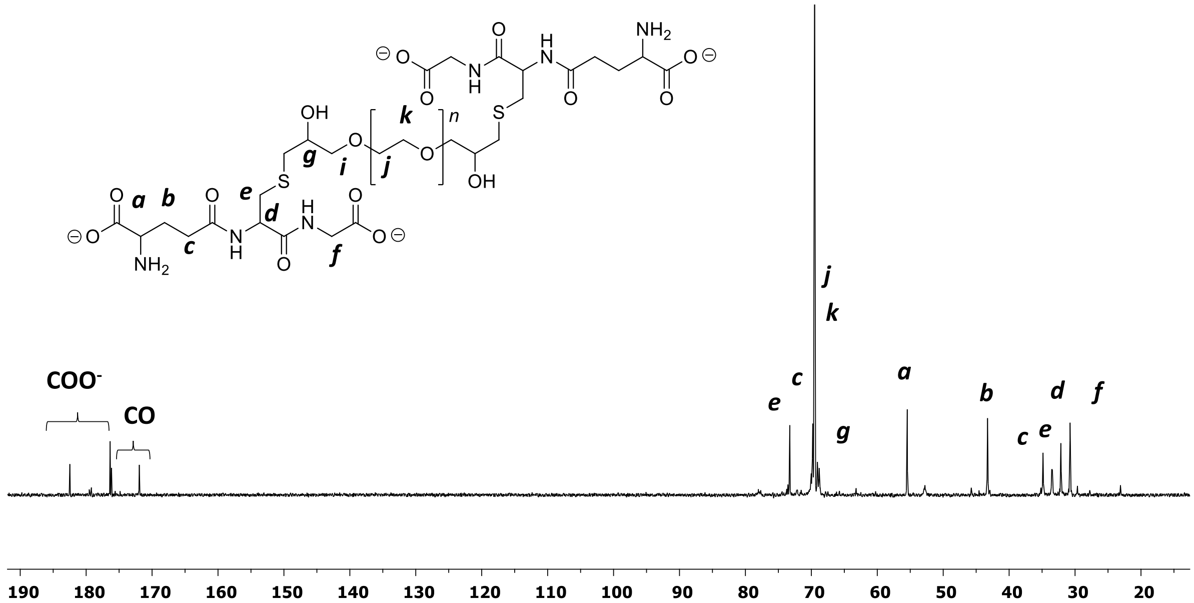

Figure S17. ^{13}C NMR of glutathione-functionalized polyethylene glycol obtained under basic conditions in D_2O at room temperature.

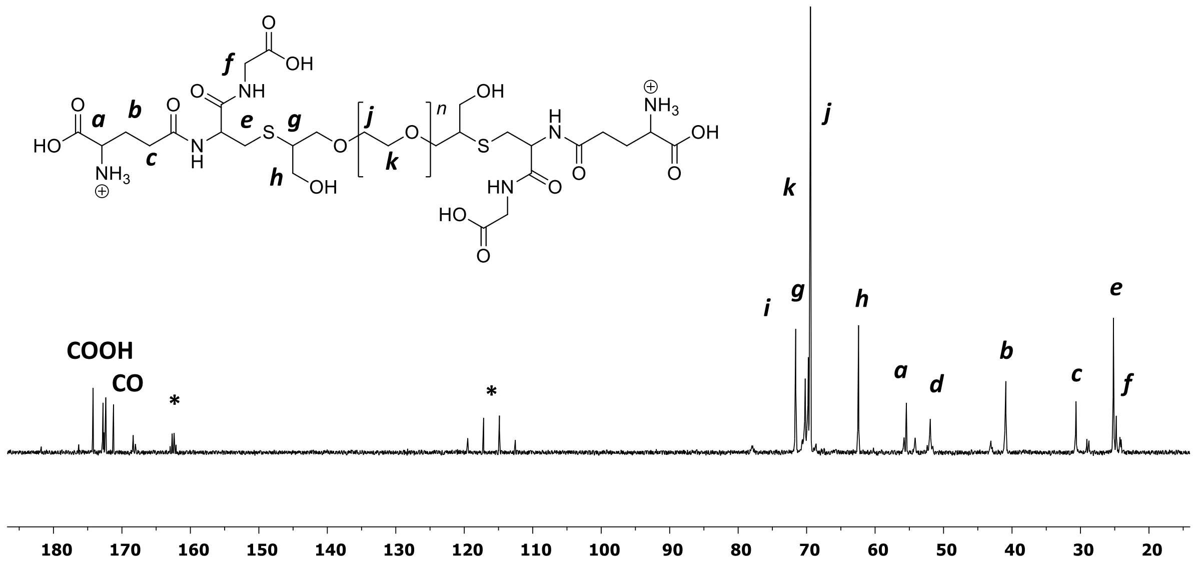

Figure S18. ^{13}C NMR of glutathione-functionalized polyethylene glycol obtained under acidic conditions in D_2O at room temperature. The signals from trifluoroacetic acid are shown with an asterisk.

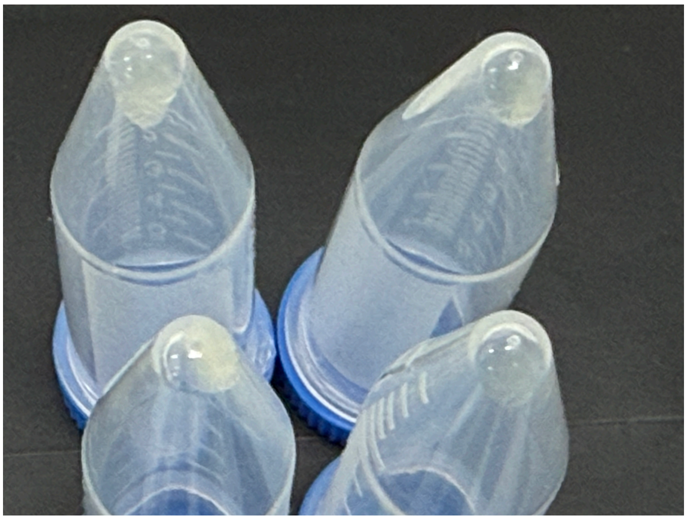

Figure S19. Digital picture of the isolated polymer in plastic centrifuge tubes.
